# Social inequalities in the effects of school-based well-being interventions: a systematic review

**DOI:** 10.1093/eurpub/ckaf005

**Published:** 2025-02-20

**Authors:** Eetu Haataja, Heidi Leppä, Mikko Huhtiniemi, Rozenn Nedelec, Tiina Soini, Timo Jaakkola, Mika Niemelä, Tuija Tammelin, Marko Kantomaa

**Affiliations:** Research Unit of Population Health (PopH), Faculty of Medicine, University of Oulu, Oulu, Finland; LIKES, Jamk University of Applied Sciences, Jyväskylä, Finland; Faculty of Sport and Health Sciences, University of Jyväskylä, Jyväskylä, Finland; Research Unit of Population Health (PopH), Faculty of Medicine, University of Oulu, Oulu, Finland; Faculty of Education and Culture, Tampere University, Tampere, Finland; Faculty of Sport and Health Sciences, University of Jyväskylä, Jyväskylä, Finland; Research Unit of Population Health (PopH), Faculty of Medicine, University of Oulu, Oulu, Finland; LIKES, Jamk University of Applied Sciences, Jyväskylä, Finland; Research Unit of Population Health (PopH), Faculty of Medicine, University of Oulu, Oulu, Finland

## Abstract

Rising public concern about comprehensive child and adolescent well-being has led to the development of school-based interventions with the potential for high-reaching and effective support. While some interventions have shown effectiveness, limited understanding exists regarding how social inequalities are considered and evidenced in such interventions. This study examines how social inequalities are considered in universal school-based interventions and their potential to affect inequalities through differential effects. A systematic review following the PRISMA protocol was conducted using the following databases: PubMed, Web of Science, CINAHL, Scopus, ProQuest and APA PsycArticles. Studies published between 2014 and 2023 were included. Screening and data extraction were conducted independently by two researchers. Of 10 028 initial articles, 44 were included in the final analysis. These studies primarily involved physical activity and mindfulness interventions in schools. Despite many studies including information regarding students’ social backgrounds, such as socioeconomic position and immigrant background, the analysis of differential intervention effects among demographic groups was limited and mostly based on sex. Most differential effect analyses showed no significant differences based on social background, and no clear differences were found based on intervention type. While some universal school-based interventions show promise in reducing social inequalities in students’ well-being, more empirical research is needed to explicitly target these questions. This review highlights the critical need for comprehensive intervention studies to consider and report relevant dimensions of social background and their interactions with intervention effects.

**Trial registration:**

PROSPERO; registration no. CRD42023423448

## Introduction

While most school students do well, an increasing number are experiencing difficulties with their well-being [1]. Well-being is often understood as an individual’s overall state of being, encompassing physical, mental and social dimensions [2]. It involves positive attributes like life satisfaction and the absence of negative states such as anxiety. It is closely related to mental health which, however, often shifts focus on the absence of mental health conditions, such as mental disorders and psychosocial disabilities [3]. In the educational context, especially, a holistic perspective is needed to recognize the multifaceted nature of well-being. In Western countries, a decline in students’ well-being has been observed since the beginning of the 21st century, particularly among teenage girls [4, 5]. For example, in Finland, the percentage of 15- to 16-year-old adolescent girls reporting their health as average or bad has increased from 19.1% to 36.1% within the last 10 years [6].

Furthermore, preliminary evidence suggests that youth well-being is becoming increasingly unequal [7, 8]. Specifically, increasing social inequalities manifest as systematic differences in well-being across socioeconomic positions, genders, ethnicities, sexual orientations or other social groups with differentiated access to material and non-material resources [9, 10]. In health inequality research, the PROGRESS-Plus framework [11] is a tool used to describe and categorize social determinants, including place of residence, race, ethnicity, culture or language, occupation, gender or sex, religion, education, socioeconomic position and social capital. However, little is known about how these dimensions of inequality highly relevant to well-being [2, 12] have been considered in intervention studies aimed at improving comprehensive well-being in schools.

From a public health perspective, schools are a potential place to reach almost all children and adolescents at an early stage of their lifespans. While subgroup-targeted interventions may have the potential, in some cases, to decrease inequalities, they also carry the risk of stigmatizing individuals [13, 14]. Several universal school interventions (preventive measures targeting an entire population) have been developed to support child and adolescent well-being in schools [15]. Previous reviews have shown some of these interventions to be effective [16], for example, promoting increased physical activity [17] or mindfulness practice [18]. However, while many of these interventions have demonstrated positive effects on mental and emotional well-being [19–21], it remains unclear whether all subgroups benefit from these interventions since only some students may show a positive effect [22, 23]. Previous studies examining inequalities in objectively measured physical activity [23] and health behaviours [24], suggest that not all students benefit equally from universal school-based interventions. Potential mechanisms proposed for such differences include, for example, the variation in the continuum of interventions relying on individual agency (individuals making the choice to act) as opposed to changes in structures (school environment, norms and practices shaping the actions of individuals) [13]. Some barriers to improvement in interventions may also apply differently to different social groups. For example, low socioeconomic position (SEP) female students have reported concerns regarding their appearance, body image and self-confidence during physical activity, which could lead to lower adherence to certain types of interventions [25]. Regarding social-emotional learning interventions often aimed at improving well-being, the content and pedagogy may not align with the lived experience of students with low SEP, resulting in lower adherence [26]. In contrast, the more advantaged student group may have less to gain in some dimensions of well-being, which could potentially result in a smaller subgroup effect for them. In some cases, interventions may even hinder the well-being of some groups of students [27, 28]. Overall, there appears to be a need to understand how social inequalities unfold in universal school-based interventions that aim to improve health and well-being [28].

In 2015, a systematic review conducted by Moore *et al.* examined the effects of universal school-based health behaviour interventions and found that interventions may either narrow, widen or have no effect on inequality in health behaviours [24]. They also emphasized the need for routine testing of intervention effects on inequality, which would help draw firmer conclusions regarding the types of interventions that affect inequality. Love *et al.* studied the equity effects of children’s physical activity interventions [23]. They found that, despite most studies reporting the baseline demographic information in detail, very few studies reported analyses of differential intervention effects on physical activity outcomes based on those characteristics. The authors further concluded that there is a lack of evidence regarding the differential effects of physical activity interventions, particularly beyond gender.

Furthermore, research regarding the differential effects of universal school-based interventions on well-being has not been systematically studied. Such research would be crucial and timely to review, as observational studies show significant differences in the levels and trajectories of well-being between genders [5], socio-economic position [29] and race or ethnicity [30]. Additionally, understanding the broader picture of well-being interventions in relation to inequality is essential; research suggests that combinations of interventions may hold specific potential for improving students' well-being [16].

### Aim

This study aims to examine how social inequalities have been taken into account in universal school-based interventions and how these interventions may potentially affect inequalities through differential effects. By doing so, it offers new insights that can inform the development of more efficient and equitable interventions in the future, along with suggestions for future research on social inequalities in students’ well-being.

Research question 1. How do school-based well-being interventions take into account the social background of children and youth?

Research question 2. Are there differences in the effects of school-based interventions on the well-being of children and youth according to their social background?

## Methods

### Study registration and protocol

This review follows the Preferred Reporting Items for Systematic Reviews and Meta-Analyses (PRISMA) statement and its equity extension in reporting (Supplementary Material, File S1). The review was registered with the International Prospective Register of Systematic Reviews (PROSPERO; registration no. CRD42023423448; Supplementary Material, File S2) available from: https://www.crd.york.ac.uk/prospero/display_record.php?ID=CRD42023423448

### Searches

PubMed, Web of Science, CINAHL, Scopus, ProQuest and APA PsycArticles were used to carry out the study search. The initial search was conducted in June 2023 and was limited to studies reported since 1.1.2014 to capture the most recent decade of research, with results limited to those available in English. When available in the database, the following filters were used: English language, peer-reviewed journal article and published within the last 10 years (01 January 2014 →).

The search terms combined keywords and controlled vocabulary subject terms (MeSH, CINAHL headings, etc.) describing the study population (e.g. child or adolescent), social inequality factors (e.g. socioeconomic position), context (school), study design (e.g. intervention or trial) and outcome (e.g. well-being). Search terms were customized for each database (see Supplementary Material, File S3 for the search terms in detail). The University of Oulu Library’s senior information specialist was consulted when creating the search terms. The searches were re-run prior to the final analysis at the end of year 2023. Additional forward and backward searches were conducted using the Scopus, Web of Science and PubMed databases (07 Novermber 2023). All relevant studies cited in the eligible studies identified during the initial search, as well as any studies citing those eligible ones, were also included in the data extraction phase.

### Screening

The included studies involved children and adolescents aged 5–16 years from general primary and secondary schools. The interventions considered could be either single or multicomponent universal interventions aimed at increasing well-being (physical, psychological or social) within a school environment during the school day. Primary or secondary outcomes related to children’s and adolescents’ well-being (physical, psychological or social) had to be reported at both baseline and post-intervention. Eligible study designs included intervention studies (Randomized controlled trials, controlled trials or pre–post studies). Observational studies without an intervention were excluded. Publications meeting the criteria were peer reviewed journal articles published in English from 1 January 2014 to 31 December 2023. In addition, the eligible studies had to analyse differential intervention effects based on social backgrounds, such as subgroup analysis by sex or modelling socioeconomic position in interactions. See the full description of the criteria in Supplementary Material, File S4.

**Figure 1.**
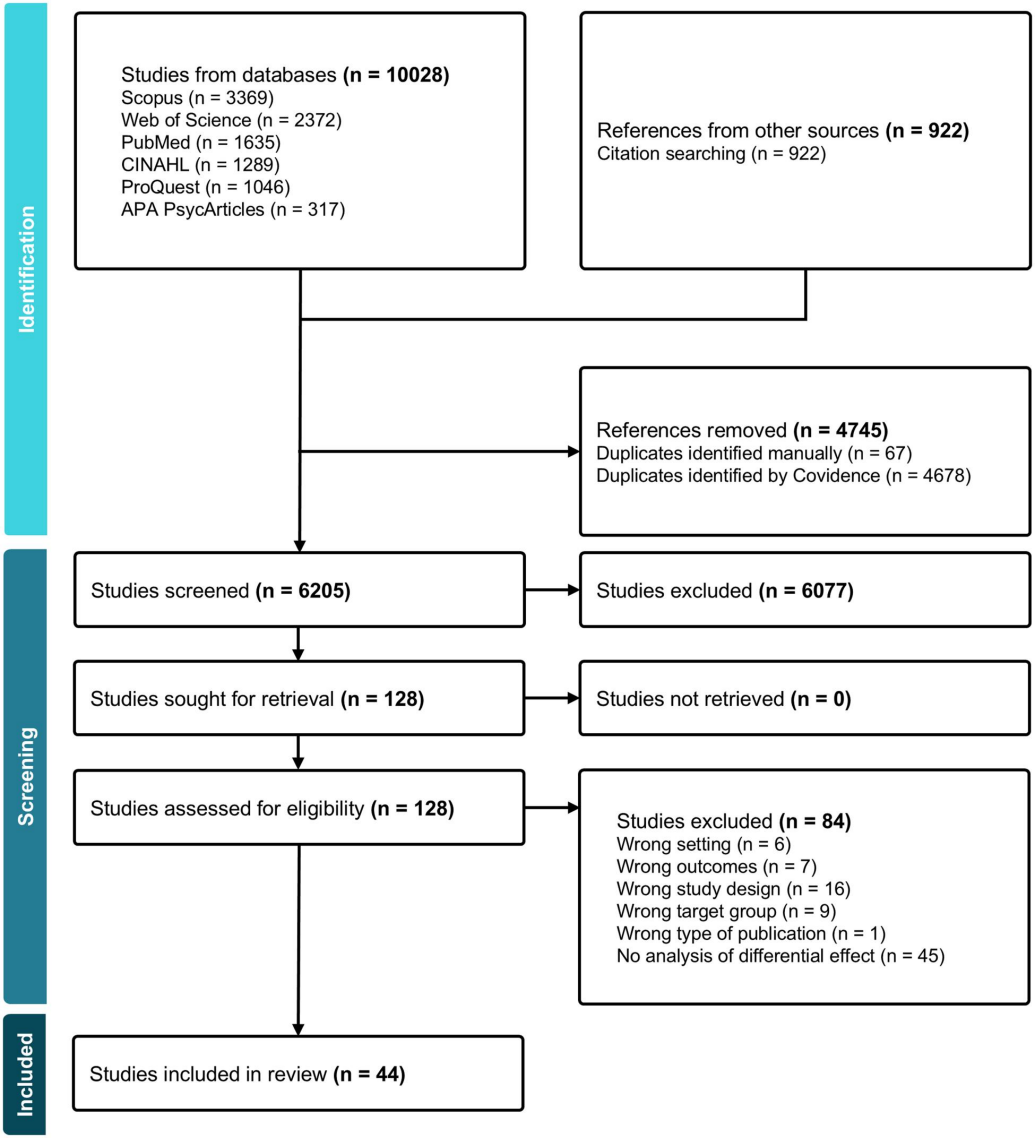
Flow diagram of the study selection process for the review. Adapted from Covidence (Covidence systematic review software, Veritas Health Innovation, Melbourne, Australia. Available at www.covidence.org).

The selected studies were added to the Covidence review management system (see Fig. 1). Two of the three researchers (E.H., H.L. and M.H.) screened each abstract independently; any disagreements were resolved through discussion between them. Afterwards, two researchers (E.H. and H.L.) examined the full texts to confirm eligibility and any disagreements were resolved through further discussion. The reasons for excluding each study were noted during the full-text phase.

### Data extraction

Two independent researchers (E.H. and H.L.) extracted data from each study included in the review, using Covidence software. The data were extracted in duplicate to ensure accuracy. The information collected from each study included the aim of the study, study design, type of intervention, start and end dates, funding sources, conflicts of interest, population description, inclusion and exclusion criteria, school level, sample size, outcome measurements, social background variables, baseline population characteristics, a description of the intervention and comparisons, outcome variables, subgroup differences before the intervention, results of the subgroup and moderation analysis and a discussion regarding social inequalities in well-being.

### Quality assessment

The quality of the included studies was evaluated using a quality assessment tool (Effective Public Health Practice Project Quality Assessment Tool for Quantitative Studies) [31]. The studies were evaluated based on their reported selection bias, study design, confounders, blinding, data collection method, withdrawals and dropouts. The first and second authors of the study initially evaluated each area individually. Subsequently, consensus was reached through negotiation to resolve any potential disagreements. Based on the evaluation results, a global rating was assigned for each study.

### Synthesis

The data collected were further analysed narratively. The collection of different social background variables was quantified and further compared with the number of variables used in the differential analysis. The differential effects of the interventions were listed by intervention type. No further disaggregation was possible, given the number of studies. The overview of the statistically significant differential effects was then visualized using Harvest plots. The bar plot and harvest plot figures were drawn using R version 4.3.2 and ggplot2 (3.5.0).

## Results

### Study characteristics

The search initially yielded a total of 10 028 articles, which were reduced to 6205 after removing duplicates. Of these, 128 studies were full-text screened. The lack of differential effect analysis was the most common reason for exclusion in this stage (*N *=* *45). After screening, 44 articles met the inclusion criteria and were included in the final analysis. Among the selected articles, 21 studies (48%) were conducted in Europe, including countries such as the UK, Denmark, Sweden, Finland, Estonia, Norway, Belgium, Austria and Portugal. Twelve studies (27%) were carried out in Australasia, which comprises Australia and New Zealand. Six studies (14%) were performed in Asia, including China, Japan, South Korea and India. Three studies (7%) were conducted in the Middle East, specifically in Israel, while two studies (5%) were carried out in North America, specifically in the USA. Furthermore, 21 studies were conducted in a primary school context, 20 studies were carried out in a secondary school context, and three studies involved both primary and secondary schools. Cluster randomized trials were the most common type of design within the studies (*N *=* *28), followed by non-randomized experimental studies (*N *=* *10) and randomized controlled trials (*N *=* *4). Two studies utilized a pretest–posttest design. In 20 of the studies, the aim and purpose involved an explicit statement of studying inequalities or differential effects. Most studies reported multiple outcomes related to well-being (*Mdn *=* *3). The list of the different categorized outcomes can be found in Supplementary Material, File S5.

The selected studies utilized multiple types of intervention, which were grouped into 11 categories. The most common intervention type was physical activity (*n *=* *8), followed by mindfulness interventions (*n *=* *6) and multimodal interventions (*n *=* *6), involving combinations of interventional elements such as physical activity and nutrition. Of the 44 studies, 14 were published between 2014 and 2018 and 30 between 2019 and 2023. The quality of 32 studies was assessed as weak, six as moderate, and six as strong (see Supplementary Material, File S6). Table 1 summarizes the main characteristics of the studies analysed in this review.

**Table 1.** Description of the included studies

Authors	Year	ID	Country	School level	Intervention (name)	Length of the intervention	General effectiveness	Sample size (school/class/individual)
Atkins and Hayes	2019	[32]	UK	Secondary	Autogenic training relaxation intervention	6 weeks	Anxiety ↘	4/NR/66
Bell *et al.*	2022	[33]	UK	Secondary	Body image intervention (digital bodies)	One session	NR	1/11/290
Borman *et al.*	2019	[34]	USA	Secondary	Social-psychological reflective writing exercises	Two writing sessions	School trust ↗	11/NR/1304
							Social belonging ↗	
							Identification with school ↗	
							Evaluation anxiety ↘	
Brennan *et al.*	2021	[35]	UK	Primary	Food environment intervention, Educational cross-curricular intervention (nourish)	6 months	∅	15/47/903
Bunketorp *et al.*	2015	[36]	Sweden	Primary	Extra physical activity in schools (school-in-motion)	NR	∅	4/NR/545
Bølling *et al.*	2019	[37]	Denmark	Primary	Education outside the classroom/pedagogical practice	School year	Prosociality ↗	18/48/911
							Hyperactivity-inattention ↘	
							Peer-problems ↘	
Carroll *et al.*	2020	[38]	Australia	Primary	Whole-of-class social-emotional learning programme (KooLKIDS whole of class programme)	13 weeks	∅ (not applicable)	6/21/524
Christiansen *et al.*	2018	[39]	Denmark	Primary	Physical activity intervention (The Move for Well-being in School study (MWS))	9 months	∅	24/144/3124
Costigan *et al.*	2016	[40]	Australia	Secondary	High-intensity interval training (aerobic exercise program, AEP)	8 weeks	Well-being ↗	1/3/65
							Perceived appearance ↗	
Diao *et al.*	2020	[41]	China	Primary Secondary	Family-individual-school-based comprehensive intervention (family-individual-school-based comprehensive intervention)	One year	Psychological QoL ↗	4/4-6/948
							Social QoL ↗	
							Pubertal QoL ↗	
							Total QoL ↗	
Ford *et al.*	2019	[42]	UK	Primary	Incredible Years^®^ Teacher Classroom Management (TCM) programme	6 Months	Total difficulties ↘	80/NR/2075
Garbett *et al.*	2021	[43]	India	Secondary	Body image intervention (Confident Me)	5 weeks	Body esteem ↗	2/5/166
							Positive affect ↗	
Golan *et al.*	2014	[44]	Israel	Secondary	Wellness programme (In Favor of Myself)	8 weeks	∅ (not applicable)	1/NR/300
Gordon *et al.*	2021	[45]	Australia	Secondary	Social media literacy program (Social media literacy program (SoMe))	4 weeks	Drive for muscularity ↗	7/67/892
Harris *et al.*	2022	[46]	New Zealand	Secondary	High-intensity interval training (HIIT)	16 weeks	∅	8/NR/368
Harvey *et al.*	2023	[47]	Australia	Secondary	Dialectical behaviour therapy (WISE Teens)	8 weeks	∅	4/37/1071
Ialongo *et al.*	2019	[48]	USA	Primary	The PAX Good Behaviour Game	School year	∅	27/NR/5611
Iwahori *et al.*	2022	[49]	Japan	Primary	Mental health programme (The Treasure File Programme)	School year	Self-esteem ↗	8/70/1718
Johnson *et al.*	2016	[50]	Australia	Primary & Secondary	Mindfulness intervention (The .B, Mindfulness in Schools curriculum)	8 weeks	∅	5/17/415
Johnson *et al.*	2017	[51]	Australia	Secondary	Mindfulness programme with parental involvement (The .B, Mindfulness in Schools curriculum)	9 weeks	∅	4/NR/555
Kiviruusu *et al.*	2016	[52]	Finland	Primary	Socio-emotional skills intervention (The Together at School program)	School year	∅	79/250/3704
Laakso *et al.*	2023	[53]	Finland	Primary	Positive Education Program (The Flourishing Students program)	School year	Negative emotions ↘	5/8/136
							Loneliness ↘	
							Calmness ↗	
							Enjoyment of being alone ↗	
Lassander *et al.*	2021	[54]	Finland	Primary Secondary	Mindfulness (Stop and Breathe)	9 weeks	HRQoL ↗	56/210/3519
Lee *et al.*	2018	[55]	South Korea	Primary	Prevention program for eating disorders (Me, You and Us)	6 weeks	Body satisfaction ↗	1/6/169
							Self-esteem ↗	
Li *et al.*	2019	[56]	China	Primary	Obesity prevention intervention (Chirpy Dragon)	One year	HRQoL ↗	40/40/1641
Lubans *et al.*	2022	[57]	Australia	Primary	Whole-of-school program (The iPLAY intervention)	4.5 years	Subjective well-being ↗	130/NR/5959
Madsen *et al.*	2020	[58]	Denmark	Primary	Physical activity and health education (11 for Health in Denmark)	11 weeks	Peers and Social support ↗	111/NR/3061
Magalhães *et al.*	2022	[59]	Portugal	Primary	Mindfulness‑Based Program	8 weeks	Emotional regulation ↗	1/4/57
Montero-Marin *et al.*	2022	[60]	UK	Secondary	The School-Based Mindfulness Training (SBMT)	Spring term	∅ (NR)	84/NR/8376
Olive *et al.*	2019	[61]	Australia	Primary	Specialist-taught PE (LOOK)	4 years	Body dissatisfaction ↘	29/68/853
							Depression (ineffectiveness) ↘	
Peltonen *et al.*	2022	[62]	Finland	Secondary	In-service teacher training, peer integration and enhancement resource (INSETT/PIER)	2 seminar days	∅	16/NR/1974
Pollak *et al.*	2023	[63]	Austria	Secondary	Socio-Emotional Learning Programme (You, Me and the Little Monsters)	6 months	Social skills ↗	NR/35/680
							Peer connectedness ↗	
							Happiness ↗	
Shinde *et al.*	2018	[64]	India	Secondary	Multi-component secondary school intervention (SEHER)	Academic year	School climate ↗	74/NR/21550
							Bullying ↘	
							Violence ↘	
							Depression ↘	
							Attitudes towards gender equity ↗	
							Knowledge of reproductive and sexual health ↗	
Shoshani *et al.*	2014	[65]	Israel	Secondary	Positive psychology intervention (Maytiv School Program)	School year	Distress ↘	2/30/1038
							Anxiety ↘	
							Depression ↘	
							Interpersonal sensitivity ↘	
							Self-esteem ↗	
							Self-efficacy ↗	
							Optimism ↗	
Shoshani *et al.*	2016	[66]	Israel	Secondary	Positive psychology program (The Maytiv program)	30 weeks	Positive emotions ↗	6/70/2517
							Peer relations ↗	
							Emotional engagement in school ↗	
							Cognitive engagement ↗	
							GPA ↗	
Skoradal *et al.*	2023	[67]	Denmark	Primary	Multifactorial health promotion program (11 for Health)	11 weeks	Physical well-being ↗	19/NR/261
							Peers and social support ↗	
Smith *et al.*	2018	[68]	Australia	Secondary	Physical activity intervention, resistance training (Resistance training for teens)	10 weeks	∅	16/NR/607
Spence *et al.*	2014	[69]	Australia	Secondary	Universal mental health intervention (BeyondBlue intervention)	3 years	Depression ↘	50/NR/5633
							Anxiety ↘	
							Well-being ↗	
Stjernqvist *et al.*	2018	[70]	Denmark	Primary	Health-promoting school intervention (We Act Together for Health)	6 months	Sense of belonging in the school ↘	8/30/595
Streimann *et al.*	2020	[71]	Estonia	Primary	The PAX Good Behaviour Game (PAX GBG))	2 years	Mental health ↗	42/42/708
Torok *et al.*	2019	[72]	Australia	Primary	PAX Good Behaviour Game	12 weeks	Emotional problems ↘	6/11/230
							Behavioural problems ↘	
Troncoso and Humphrey	2021	[73]	UK	Primary	Behavioural intervention (The Good Behaviour Game)	2 years	Prosocial behaviour ↗	77/279/3084
							Concentration problems ↘	
Volkaert *et al.*	2022	[74]	Belgium	Secondary	Emotion regulation program	6 months	Depressive symptoms ↘	6/NR/347
							Self-esteem ↗	
							Indirect bullying experiences ↘	
							Psychological stigmatization ↘	
Åvitsland *et al.*	2020	[75]	Norway	Secondary	Physical activity intervention, active learning	29 weeks	∅	29/NR/2084

### Measures of social background and usage

**Figure 2.**
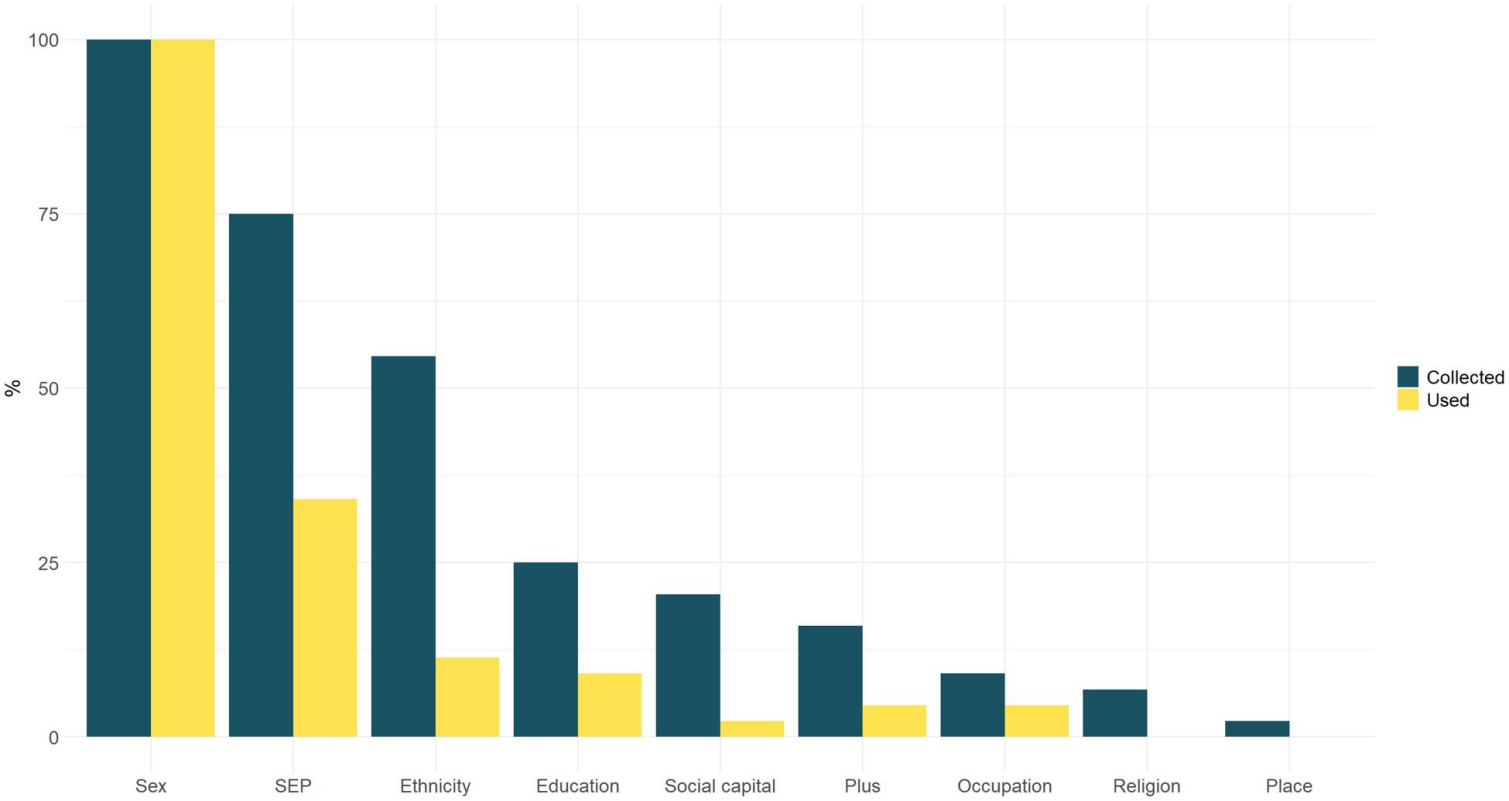
Percentage of relevant studies collecting PROGRESS plus measures vs. percentage using those for differential analysis. ‘Plus’ refers to additional context-specific factors.

Figure 2 shows the proportions of social background measures collected in the studies. All 44 studies included information about the participants’ sex or gender. Thirty-three of the 44 studies collected some form of information regarding SEP. The operationalization of SEP varied between the studies. The most common dimensions to determine SEP were family economic status, free or reduced lunch price and school area SEP indices. Six studies utilized multiple SEP dimensions simultaneously.

Although many studies collected multiple social background variables aligned with the PROGRESS-Plus equity framework, most studies utilized only a single dimension to test for differential effects (*Mdn *=* *1). Seventeen studies used several collected variables in the subgroup analysis. The maximum number of social background variables used for differential analysis in a single study was 5. With multiple outcomes, this resulted in 24 statistical tests. The most common background variables used in the subgroup analysis were sex (*n *=* *44) and SEP (*n *=* *15). However, ethnicity, the third most commonly used subgrouping variable, was included in the analysis only five times. Variables from the ’plus’ section of the PROGRESS-Plus framework, such as discrimination and family support, were each used only once in the subgroup analysis.

### Differential intervention effects

**Figure 3.**
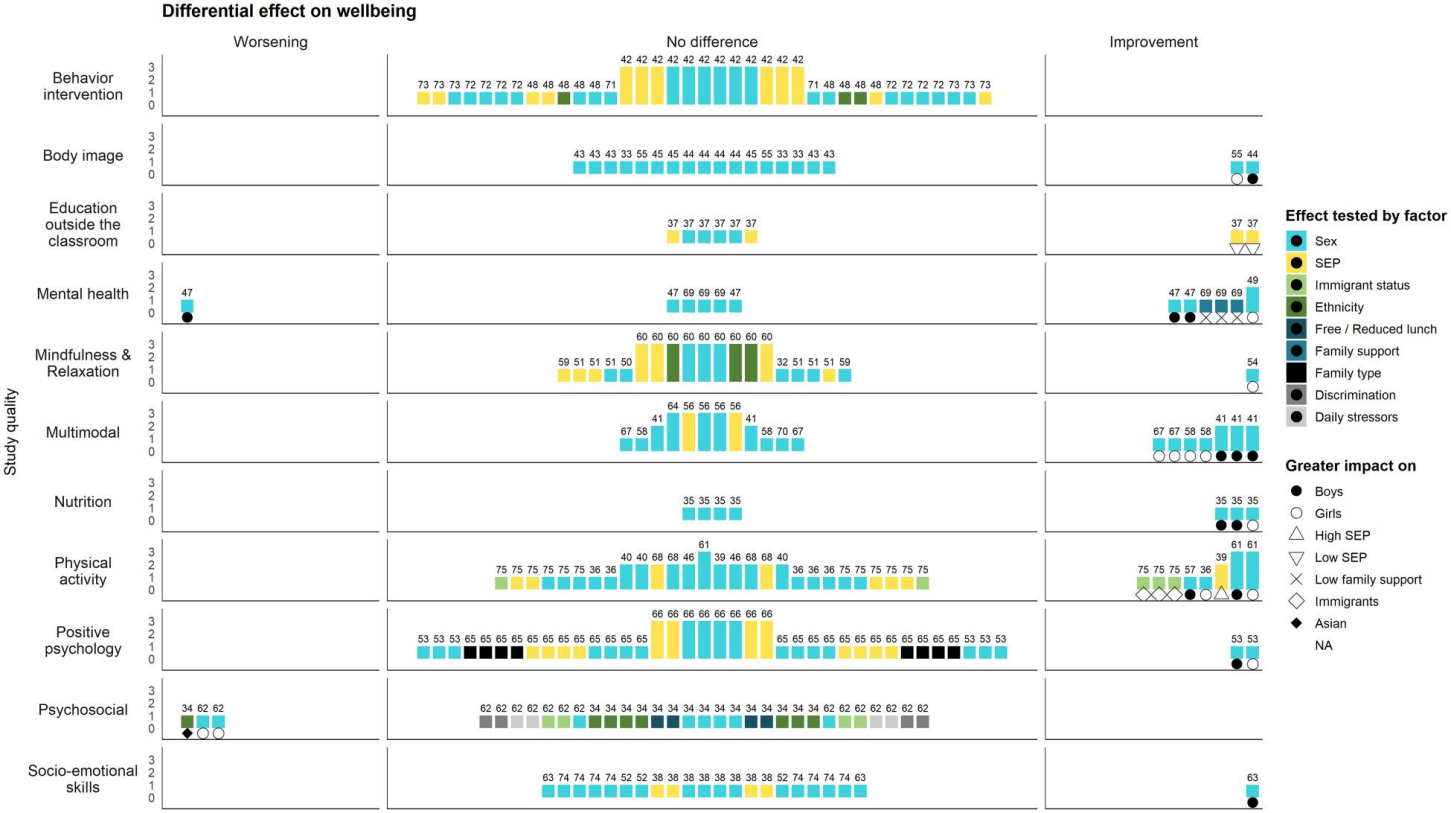
A Harvest plot showing differential improvement and worsening effects reported in the studies. Bars under the ‘improvement’ column indicate significant differential effects, with subgroups (denoted by symbols) benefiting more from the intervention. Bars under the ‘worsening’ column indicate stronger negative effects for the specified subgroups. Bar height represents study quality, and the number above each bar corresponds to the review reference number.

In the 44 eligible studies, 252 differential effects moderated by social background were statistically tested (see Fig. 3). Most of the differential effects did not show statistical significance. Overall, 32 differential improvement effects were found in eight studies. Most of the differential positive effects occurred due to sex, where boys benefited more from the intervention in 12 tested effects and girls in 11 tested effects. Two studies identified differential effects based on SEP. One study found two positive effects for education outside the classroom intervention, especially for students with low SEP backgrounds. In contrast, one study reported that a physical activity intervention had a more profound effect on high SEP students. Four effects from the three studies were found to have differentially negative outcomes.

Overall, most of the studies did not provide information on either baseline subgroup differences in well-being or the numeric estimates of effect sizes of differential effects. Including this combination would allow for a more detailed interpretation of changes in inequalities. Six studies provided some evidence of a decrease in inequality, while in two studies, inequalities appeared to have increased.

## Discussion

This study aimed to review the information collected on various social background dimensions and how it was used to analyse the effects of school-based interventions on well-being. Additionally, the study sought to identify whether the intervention effects differed based on social background. The results show that many of the dimensions in the PROGRESS-Plus framework are covered in the studies’ social background information. However, only a few of the collected variables were used to analyse the differential effects of the interventions. Notably, traditional variables, such as sex and SEP, are predominately used for differential effect analysis, often without an explicit rationale. These findings align with prior reviews [24, 76], underscoring a general neglect of social inequalities in universal school-based health intervention studies. This neglect leads to scarce evidence regarding the differential impact of interventions according to child or adolescent social backgrounds. So far, most of the studies have been conducted in Europe, with a slight increase in the number of studies during the last 10 years.

Most tested effects did not show differences based on students’ social backgrounds. Although some studies hinted at more favourable outcomes for certain social groups, a clear signal favouring any specific social group was not evident, largely due to significant effects being found across multiple social categories. The reason for the rather low number of scattered differential effects could be the study sample sizes, which were probably not determined in most cases to meet the demands of a more complex subgroup analysis. Furthermore, aligning with prior inequality-themed reviews [23], studies providing more specific interpretations of changes in inequalities were scarce, meaning that the pre- and post-intervention statistics stratified by social background were often not reported. However, it is encouraging that multiple studies clearly aimed to explore social inequalities. Such aims, coupled with recent suggestions [28] on how to address inequalities at each step of the universal school-based intervention research (i.e. in the provision of and access to interventions and resources, uptake, efficacy and long-term compliance), provide an important stepping stone for future research.

Furthermore, the literature has increasingly acknowledged the complexity of the effects that different social positions can have, particularly at their specific intersections (e.g. girls with ethnic minority backgrounds). It should be studied further within intervention studies [77]. In this review, two studies [34] tested for such three-way interaction effects, addressing the ethnicity dimension at the intersection. The challenge lies in the additional demands for sample size and research design as the models become more complex. Still, evidence regarding intervention effects for such vulnerable groups is crucial, as they stand to benefit the most from reducing social inequalities in comprehensive well-being [10].

The findings of this systematic review have important implications for researchers, practitioners and policymakers. For researchers, the study highlights the need for a more comprehensive and rigorous investigation of social background dimensions within universal school-based well-being interventions. There is a need to consider potential equality effects with the PROGRESS-Plus perspective prior to intervention and to take those dimensions into account, for example, in relation to sample size and pre-specified hypothesized subgroup effects [78]. We concur with the recommendations of prior reviews [23, 76] and encourage researchers to provide information regarding the sample size, mean and standard deviation of well-being at baseline and subsequent follow-ups for both intervention and control groups, for the main intervention effect and stratified by the social background variable of interest (i.e. SEP). Later on, this would allow the accumulation of differential effect analysis and adequate statistical power to answer the questions regarding intervention inequality. Another potential approach to tackling some of the challenges related to sample size could be large natural experiments. They utilize the fact that real-world policies or interventions often involve an arbitrary cutoff, assigning people to ‘intervention’ and ‘control’ groups almost randomly [79].

Practitioners implementing school-based interventions should be aware of the potential differential effects on various student subgroups. This review provides preliminary evidence and examples suggesting that both large-scale whole-of-school interventions [69] and small-scale adjustments to pedagogical practices [37], can have the potential to reduce inequalities. A study reported a decrease in SEP-based inequalities after implementing a change in daily pedagogical practices involving weekly teaching conducted outside the classroom throughout the school year [37]. An efficient benefit of such pedagogy-targeted interventions is their possible integration into curriculum-based learning content. In this particular case, the teachers received 2 days of pedagogical training, which, from the policymaker's perspective, emphasizes the potential of teacher training and teacher continuing education as large-scale pathways for improving well-being in schools successfully. Such an approach is supported by research in which teacher professional development has shown the potential to reduce the inequality gaps in learning [80]. For researchers, this type of setting could simultaneously afford large natural experiments with larger sample sizes and higher certainty in interpreting differential effects [79].

While this review found limited evidence to support any particular type of intervention in reducing social inequalities in student well-being, discussions regarding the factors necessary for reducing inequalities in well-being interventions are emerging in the research literature. For example, based on their observations and experience, Mansfield *et al.* recently stated that critical priorities to achieve inclusive, engaging and inequality-reducing interventions include addressing multiple determinants of well-being, tailoring the intervention to the school context and maximizing inclusive engagement, through co‐production with students, parents and school staff [81]. Hopefully, accumulating research will demonstrate the effectiveness of these approaches in reducing social inequalities in school-based well-being interventions.

### Strengths and limitations of the review

This review has several strengths, such as the comprehensive use of multiple databases, PROSPERO pre-registration and the fact that the screening and data extraction for each study was done by two researchers independently. This study also has several limitations. For example, as the study had a broad bird’s-eye view of the interventions improving well-being, the diversity and limited number of studies did not allow firm conclusions regarding the effects of inequalities. Although the search strategy focused on studies explicitly mentioning ‘well-being’, this might have excluded relevant research using alternative concepts, such as ‘mental health’. Furthermore, one quality assessment tool may not capture all aspects relevant to different research designs. While efforts were made to analyse differential intervention effects based on social background variables, the review found limited reporting and analysis of such effects in the included studies. This limitation hindered the comprehensive assessment of how interventions impact different social groups and their potential to address social inequalities in well-being. Most of the studies were carried out in Europe, which may also restrict the generalisability of the results to other areas. Despite these limitations, the review provides valuable insights into the current state of research regarding social inequalities in school-based well-being interventions.

## Supplementary Material

ckaf005_Supplementary_Data

## Data Availability

No new data were generated or analysed in this study. All data supporting the findings of this systematic review are derived from previously published studies and are available in the public domain. Key points School-based well-being interventions are widely implemented but often overlook the impact of social inequalities. The majority of included studies focused on interventions based on physical activity and mindfulness. There is limited evidence of differential effects by social background, with most studies focusing primarily on sex differences. More comprehensive research is needed to understand how these interventions affect various social groups. There is preliminary evidence that both large-scale whole-of-school interventions and small-scale adjustments to pedagogical teaching practices could potentially reduce inequalities.
