# Supplementary material for: Social inequalities in the effects of school-based well-being interventions: a systematic review
Source: Eur J Public Health. 2025 Feb 20;35(2):302–11. doi: 10.1093/eurpub/ckaf005 (PMC11967906; doi:10.1093/eurpub/ckaf005)
Supplement: ckaf005_Supplementary_Data [file ckaf005_supplementary_data.zip › ckaf005_Supplementary_Data/ejph-2024-05-om-0341-File005.pdf]

Social inequalities in the effects of school-based well-being interventions: a systematic review

To enable PROSPERO to focus on COVID-19 submissions, this registration record has undergone basic automated checks for eligibility and is published exactly as submitted. PROSPERO has never provided peer review, and usual checking by the PROSPERO team does not endorse content. Therefore, automatically published records should be treated as any other PROSPERO registration. Further detail is provided here.

Citation

Eetu Haataja, Heidi Leppä, Mikko Huhtiniemi, Rozenn Nedelec, Tiina Soini-Ikonen, Timo Jaakkola, Mika Niemelä, Tuija Tammelin, Marko Kantomaa. Social inequalities in the effects of school-based well-being interventions: a systematic review. PROSPERO 2023 CRD42023423448 Available from: https://www.crd.york.ac.uk/prospero/display_record.php?ID=CRD42023423448

Review question

We investigate social inequalities in children and youth well-being in school-based interventions.

RQ1. To which extent school-based well-being interventions take into account the social background of children and youth?

RQ2. Are there differences in the effects of school-based interventions on children and youth well-being according to their social background?

RQ3. Which components and characteristics of social background may explain these differences?

Searches

We will search the following databases: PubMed, Scopus, CINAHL (EBSCO), Web of Science, ProQuest Central, and Google Scholar. In addition to the search from databases, we will supplement the search manually by searching relevant articles from the reference lists of the eligible studies. When available, the following filters will be used in the databases: English language, peer-reviewed journal article, published within the last 10 years (01.01.2014 ->). We will re-run the searches prior to the final analysis.

The search terms combine keywords and controlled vocabulary subject terms (MeSH, CINAHL Headings etc.) describing the study population (e.g., child or adolescent), social inequality factors (e.g., socioeconomic status), context (school), study design (e.g., intervention or trial), and outcome (e.g., wellbeing). Search terms are customized for each database. The University of Oulu Library Informatician was consulted when creating the search terms. Examples of the used search terms are presented in Table 1.

Types of study to be included

We will include randomized controlled trials, quasi-randomized controlled trials, controlled before-and-after studies, prospective cohort studies, and qualitative studies.

Condition or domain being studied

Studies that investigate the effects of school-based interventions on students' well-being.

Participants/population

Primary and secondary school-aged children and adolescents (5 - 16 years).

Intervention(s), exposure(s)

The review includes all types of interventions which are aiming to increase well-being (physical, mental, social) in a school setting. Intervention can have multiple components, such as environmental changes or regulations that promote behaviors relevant to well-being.

Comparator(s)/control

The control group should come from the same population or should be matched on key factors. Interventions can be compared with a control intervention (standard or usual care) or minimal intervention control group.

Context

Studies conducted in the primary or secondary school setting are included.

Main outcome(s)

Change in different measures of well-being (physical, mental, social). The measures can involve subjective and objective components.

Additional outcome(s)

None.

Data extraction (selection and coding)

We will conduct the systematic review in accordance with the preferred reporting items for systematic review and meta-analysis (PRISMA) guidelines (Page et al. 2021). After the execution of the database search, the titles and abstracts of studies will be imported to Covidence. Duplicates will be removed, and two reviewers will independently screen the titles and abstracts of the identified studies based on the inclusion and exclusion criteria. After screening 40 articles, the reviewers will discuss the disagreements and eligibility criteria and adjust the standards if needed. After screening and finding the potential studies both reviewers will independently read the full texts and asses the eligibility.

From the selected studies, authors will extract the following information when available:

• Study design: measurement points, relevant well-being outcomes, instruments

• Study characteristics: citation, authors, publication year, the study objective

• Population characteristics: age, sample size, school type, country

• Intervention: aim, type, duration, year(s)

• Control group: the content of the treatment

• Details of social (background) factors (e.g. SES), measures collected, and how these were used

• Findings on social inequalities in the impacts of interventions targeting well-being

o To what extent did the interventions take into account the social background of children and youth?

o Were there differences in the intervention effects according to the social background?

o Which components/characteristics of social background may explain these differences?

• Main findings and conclusions

• Other discussions regarding social inequalities

After independent work, the extracted data will be compared. In case of missing information, the corresponding author of the original study will be contacted. In case of disagreements (screening the articles, data extraction) between individual judgments, the third reviewer is consulted, and the final decision is made together.

Risk of bias (quality) assessment

The quality of the included studies will be evaluated with a quality assessment tool (e.g., Effective Public Health Practice Project (EPHPP) Quality Assessment Tool for Quantitative

Studies or Cochrane risk of bias tool 2 (RoB 2)).

Strategy for data synthesis

A summary of the findings from the included studies concerning the type of intervention, intervention content, population, and reported outcomes will be provided in tables. Social inequalities in the effects of well-being interventions will be listed by intervention and outcome type, inequality factor type (i.e. family level measures such as parental education, occupation or income, or school-level measures such as area income), and by main effect (i.e. whether or not the intervention was reported to have a significant aggregate effect). In addition, narrative methods will be used to summarize the results, which can differ depending on the context.

Analysis of subgroups or subsets

Not applicable

Contact details for further information

Eetu Haataja

eetu.haataja@oulu.fi

Organisational affiliation of the review

Research Unit of Population Health, Faculty of Medicine, University of Oulu, Finland

www.oulu.fi

Review team members and their organisational affiliations [1 change]

Dr Eetu Haataja. Research Unit of Population Health, Faculty of Medicine, University of Oulu

Dr Heidi Leppä. School of Health and Social Studies, Jamk University of Applied Sciences

Dr Mikko Huhtiniemi. Faculty of Sport and Health Sciences, University of Jyväskylä

Dr Rozenn Nedelec. Research Unit of Population Health, Faculty of Medicine, University of Oulu

Dr Tiina Soini-Ikonen. tiina.soini-ikonen@tuni.fi

Dr Timo Jaakkola. Faculty of Sport and Health Sciences, University of Jyväskylä

Dr Mika Niemelä. Research Unit of Population Health, Faculty of Medicine

Dr Tuija Tammelin. Likes, School of Health and Social Studies, Jamk University of Applied Sciences

Dr Marko Kantomaa. Research Unit of Population Health, Faculty of Medicine

Type and method of review

Systematic review

Anticipated or actual start date

17 March 2023

Anticipated completion date [1 change]

31 May 2024

Funding sources/sponsors

This systematic review is prepared within the project SchoolWell, funded by the Strategic Research Council within the Academy of Finland (353362 (M.K.), 353361 (T.T.).

Grant number(s)

State the funder, grant or award number and the date of award

Academy of Finland (353362 (M.K.), 353361 (T.T.).

Conflicts of interest

Language

English

Country

Finland

Stage of review [1 change]

Review Ongoing

Subject index terms status

Subject indexing assigned by CRD

Subject index terms

Adolescent; Child; Humans; NSC 30705; Schools; Socioeconomic Factors

Date of registration in PROSPERO

21 May 2023

Date of first submission

10 May 2023

Stage of review at time of this submission [1 change]

Stage	Started	Completed
Preliminary searches	Yes	Yes
Piloting of the study selection process	Yes	Yes
Formal screening of search results against eligibility criteria	Yes	Yes
Data extraction	Yes	Yes
Risk of bias (quality) assessment	Yes	Yes
Data analysis	Yes	Yes

Revision note

The author information and review progress was updated.

The record owner confirms that the information they have supplied for this submission is accurate and complete and they understand that deliberate provision of inaccurate information or omission of data may be construed as scientific misconduct.

The record owner confirms that they will update the status of the review when it is completed and will add publication details in due course.

Versions

21 May 2023
21 May 2023
17 May 2024
