# Supplementary material for: Social inequalities in the effects of school-based well-being interventions: a systematic review
Source: Eur J Public Health. 2025 Feb 20;35(2):302–11. doi: 10.1093/eurpub/ckaf005 (PMC11967906; doi:10.1093/eurpub/ckaf005)
Supplement: ckaf005_Supplementary_Data [file ckaf005_supplementary_data.zip › ckaf005_Supplementary_Data/ejph-2024-05-om-0341-File006.pdf]

Supplementary File S3

Social inequalities in the effects of school-based wellbeing interventions: a systematic review,

Haataja, Leppä, Huhtiniemi, Nedelec, Soini-Ikonen, Jaakkola, Niemelä, Tammelin, Kantomaa

Database	Study population	Social inequality factors	Context	Study design	Outcome
**General search term**	Child* OR<br>Teen* OR<br>Adolescent* OR<br>Youth OR<br>Student*<br><br>**AND**	sociodemographic OR<br>socioeconomic* OR<br>“socio economic*” OR<br>“social capital” OR<br>“social conditions” OR<br>“parental edu*” OR<br>“parental occupation” OR<br>“parental income” OR<br>“home environment” OR<br>family OR<br>poverty OR<br>neighborhood OR<br>“social environment” OR<br>inequalit* OR<br>equalit* OR<br>equit* OR<br>inequit* OR<br>disparit* OR<br><br>**AND**	school*<br>**AND**	intervention* OR<br>promot* OR<br>program* OR<br>prevent* OR<br>trial* OR<br>experiment* OR<br>random*<br><br>**AND**	wellbeing OR<br>well-being OR<br>"well being"
**Pubmed**	Child [MeSH Terms] OR<br>Child* [Text Word] OR<br>Teen* [Text Word] OR<br>Adolescent [MeSH Terms] OR<br>Adolescen* [Text Word] OR<br>Youth [Text Word] OR<br>Student* [Text Word]<br><br>**AND**	"Family Characteristics"[MeSH Terms] OR<br>"Sociological Factors"[MeSH Terms] OR<br>"Social Environment"[MeSH Terms] OR<br>"Socioeconomic Factors"[MeSH Terms] OR<br>"Health Equity"[MeSH Terms] OR<br>sociodemographic*[tw] OR<br>socio-demographic*[tw] OR<br>socioeconomic* [Text Word] OR<br>“socio economic*” [Text Word] OR<br>“parental edu*” [Text Word] OR<br>“parental occupation*” [Text Word] OR<br>“parental income*” [Text Word] OR<br>famil*[Text Word] OR<br>povert*[Text Word] OR<br>inequalit* [Text Word] OR<br>equalit* [Text Word] OR<br>equit* [Text Word] OR<br>Inequit* [Text Word] OR<br>disparit* [Text Word]<br><br>**AND**	("Schools"[MeSH Terms:noexp] OR<br>"school*"[Text Word])<br><br>**AND**	(intervention* [Text Word] OR<br>promot* [Text Word] OR<br>program* [Text Word] OR<br>prevent* [Text Word] OR<br>trial* [Text Word] OR<br>experiment* [Text Word] OR<br>random* [Text Word])<br><br>**AND**	("Psychological Well-Being"[MeSH Terms] OR<br>wellbeing [Text Word] OR<br>well-being [Text Word] OR<br>"well being"[Text Word])
**Scopus**	(Child* OR<br>Teen* OR<br>Adolescent* OR<br>Youth OR<br>Student*)	(sociodemographic* OR<br>socio-demographic* OR<br>socioeconomic* OR<br>“socio economic*” OR	school*<br>**AND**	(intervention* OR<br>promot* OR<br>program* OR<br>prevent*	(wellbeing OR<br>well-being OR<br>"well being")

	**AND**	“social capital” OR<br>“social condition*” OR<br>“parental edu*” OR<br>“parental occupation*” OR “parental income*” OR<br>“home environment” OR<br>famil* OR<br>povert* OR<br>neighborhood* OR<br>“social environment*” OR<br>inequalit* OR<br>equalit* OR<br>equit* OR<br>Inequit* OR<br>disparit*)<br><br>**AND**		OR<br>trial*<br>OR<br>experiment*<br>OR<br>random*)<br><br>**AND**	**AND NOT**<br><br>"systematic review" OR "meta-analysis" OR "mice" OR "rats"
**CINAHL**	(MH "Child+") OR (MH "Minors (Legal)") OR child* OR (MH "Adolescence+") OR adolescen* OR teen* OR youth<br><br>**AND**	(MH "Family Characteristics+") OR (MH "Social Environment+") OR (MH "Socioeconomic Factors+") OR (MH "Health Inequities") OR<br><br>sociodemographic* OR<br>socio-demographic* OR<br>socioeconomic* OR<br>“socio economic*” OR<br>“social capital” OR<br>“social condition*” OR<br>“parental edu*” OR<br>“parental occupation*” OR “parental income*” OR<br>“home environment” OR<br>famil* OR<br>povert* OR<br>neighborhood* OR<br>“social environment*” OR<br>inequalit* OR<br>equalit* OR<br>equit* OR<br>inequit* OR<br>disparit*<br><br>**AND**	school*<br><br>**AND**	(MH "Experimental Studies+") OR<br>intervention* OR<br>promot* OR<br>program* OR<br>prevent*<br>OR<br>trial*<br>OR<br>experiment*<br>OR<br>random*<br><br>**AND**	(MH "Psychological Well-Being") OR wellbeing OR<br>well-being OR<br>"well being"
**APA PsycArticles**	Child* OR<br>Teen* OR<br>Adolescent* OR<br>Youth OR<br>Student*<br><br>**AND**	(DE "Family Background") OR (DE "Family Background" OR DE "Family Socioeconomic Status" OR DE "Parent Educational Background" OR DE "Parental Occupation") OR<br>DE "Social Equality" OR DE "Gender Equality" OR DE "Gender Gap"<br>OR<br>DE "Social Equity" OR DE "Fairness" OR DE "Resource Allocation" OR<br>(DE "Social Equity" OR DE "Fairness" OR DE	school*<br><br>**AND**	DE "Intervention" OR DE "Crisis Intervention" OR DE "Early Intervention" OR DE "Family Intervention" OR DE "Group Intervention" OR DE "School Based Intervention" OR<br>intervention* OR<br>promot* OR<br>program* OR	DE "Well Being" OR DE "Employee Well Being" OR DE "Spiritual Well Being" OR DE "Subjective Well Being" OR wellbeing OR well-being OR “well being”<br><br>**AND NOT**<br><br>"systematic review" OR

Supplementary File S3

		"Resource Allocation") AND (DE "Socioeconomic Factors" OR DE "Economic Disadvantage" OR DE "Economic Resources" OR DE "Employment Status" OR DE "Income Level" OR DE "Social Class" OR DE "Social Disadvantage" OR DE "Socioeconomic Status" OR DE "Socioeconomic Status" OR DE "Family Socioeconomic Status" OR DE "Income Level" OR DE "Social Class") OR (DE "Equity" OR DE "Health Disparities" OR DE "Pay Equity" OR DE "Racial Disparities" OR DE "Social Equity") OR<br><br>sociodemographic* OR socio-demographic* OR<br>socioeconomic* OR<br>“socio economic*” OR<br>“social capital” OR<br>“social condition*” OR<br>“parental edu*” OR<br>“parental occupation*” OR “parental income*” OR<br>“home environment” OR<br>famil* OR<br>povert* OR<br>neighborhood* OR<br>“social environment*” OR<br>inequalit* OR<br>equalit* OR<br>equit* OR<br>Inequit* OR<br>disparit*<br><br>**AND**		prevent*<br>OR<br>trial*<br>OR<br>experiment*<br>OR<br>random*<br><br>**AND**	"meta-analysis" OR "mice" OR "rats"
**ProQuest**	Noft<br>(Child* OR<br>Teen* OR<br>Adolescent* OR<br>Youth OR<br>Student*)<br><br>**AND**	Noft (sociodemographic* OR<br>socio-demographic* OR<br>socioeconomic* OR<br>“socio economic*” OR<br>“social capital” OR<br>“social condition*” OR<br>“parental edu*” OR<br>“parental occupation*” OR “parental income*” OR<br>“home environment” OR<br>famil* OR<br>poverty OR<br>neighborhood* OR<br>“social environment*” OR<br>inequalit* OR<br>equalit* OR<br>equit* OR<br>Inequit* OR	Noft (school*)<br>**AND**	Noft (intervention* OR<br>promot* OR<br>program* OR<br>prevent*<br>OR<br>trial*<br>OR<br>experiment*<br>OR<br>random*)<br><br>**AND**	Noft (wellbeing OR<br>well-being OR<br>"well being")<br><br>**NOT**<br><br>"systematic review" OR<br>"meta-analysis" OR "mice" OR<br>"rats"

Supplementary File S3

		disparit*<br><br>**AND**			
**Web of Science**	(Child* OR<br>Teen* OR<br>Adolescent* OR<br>Youth OR<br>Student*)<br>(topic)<br>**AND**	sociodemographic* OR socio-demographic* OR<br>socioeconomic* OR<br>"socio economic*" OR<br>"social capital" OR<br>"social condition*" OR<br>"parental edu*" OR<br>"parental occupation*" OR "parental income*" OR<br>"home environment" OR<br>famil* OR<br>poverty OR<br>neighborhood* OR<br>"social environment*" OR<br>inequalit* OR<br>equalit* OR<br>equit* OR<br>Inequit* OR<br>disparit*<br><br>**AND**	school*<br>**AND**	intervention* OR<br>promot* OR<br>program* OR<br>prevent*<br>OR<br>trial*<br>OR<br>experiment*<br>OR<br>random*<br><br>**AND**	(wellbeing OR<br>well-being OR<br>"well being")
