# Supplementary material for: Social inequalities in the effects of school-based well-being interventions: a systematic review
Source: Eur J Public Health. 2025 Feb 20;35(2):302–11. doi: 10.1093/eurpub/ckaf005 (PMC11967906; doi:10.1093/eurpub/ckaf005)
Supplement: ckaf005_Supplementary_Data [file ckaf005_supplementary_data.zip › ckaf005_Supplementary_Data/ejph-2024-05-om-0341-File007.pdf]

Supplementary File S4

Study inclusion and exclusion criteria

	Included	Excluded
Population	Children and adolescents aged 5 to 16 years	Pre-school children, clinical population, children selected on the basis of having a specific disease or special needs
Context	General primary and secondary schools	Special need schools, special education, not school-related
Intervention	Single or multicomponent universal interventions that aimed to increase wellbeing (physical, psychological, or social) in a school environment	The intervention is not aiming to increase wellbeing
Outcomes	Primary or secondary outcomes reported at both baseline and post-intervention, children's and adolescent's wellbeing (physical, psychological or social)	Outcomes related to cognition, academic performance
Study design	Intervention studies (RCTs, CT's or Pre-Post studies)	Observational study designs without an intervention
Publication type, year, and language	Peer-reviewed journal article, published in English from 1.1.2014	Conference abstract, study protocol, report, book, published before 1.1.2014, all other languages
Analysis	The study has investigated the inequalities in the intervention effects (i.e. sub group analysis by sex, or modelling SES in interactions)	The study has not investigated any subgroup effects (i.e. controlling for SES is not enough.)
