# Supplementary material for: Social inequalities in the effects of school-based well-being interventions: a systematic review
Source: Eur J Public Health. 2025 Feb 20;35(2):302–11. doi: 10.1093/eurpub/ckaf005 (PMC11967906; doi:10.1093/eurpub/ckaf005)
Supplement: ckaf005_Supplementary_Data [file ckaf005_supplementary_data.zip › ckaf005_Supplementary_Data/ejph-2024-05-om-0341-File008.pdf]

Supplementary File S5

List of the wellbeing outcomes measured in the studies:

Mental Wellbeing:

Adaptive Emotion Regulation

Annoyed

Anxiety

Calm

Depression

Distress

Emotional Awareness

Emotional Problems

Emotional Regulation

Emotions

Happiness

Joyful

Life Satisfaction

Nervous

Optimism

Positive Affect

Positive Emotions

Psychological Distress

Sad

Stress

Worried

Quality of Life

Social Wellbeing:

Cooperation

Empathy

Horizontal Social Capital

Supplementary File S5

Interpersonal Sensitivity

Peer Connectedness

Peer Problems

Peer Relations

Peer Relationships

Peers and Social Support

Prosociality

Social Acceptance

Social Belonging

Social Competence

Social-Emotional Skills

Social-Emotional-Behavioral Functioning

Subjective Well-being

Physical Wellbeing:

Athletic Ideal Internalization

Body Dissatisfaction

Body Esteem

Body Image

Body Satisfaction

Drive for Thinness

Eating Disorders

General Health Related Quality of Life

Health Related Quality of Life

Physical Quality of Life

Physical Self-Concept

Physical Self-Worth

Physical Wellbeing

Pubertal Quality of Life

Supplementary File S5

Risk of Depression

Self-Objectification

Self-Worth by Appearance

Self-Worth by Others

Total Quality of Life

Behavioral Wellbeing:

Behavior

Conduct Problems

Disruptive Behavior

Concentration Problems

Drive for Thinness

Externalizing

Hyperactivity-Inattention

Internalizing

Peer Connectedness

Peer Problems

Peer Relations

Peer Relationships

Peers and Social Support

Psychological Adjustment

Psychological Distress

Psychological Quality of Life

Psychological Wellbeing

School Environment

School Trust

Self-Efficacy

Self-Esteem

Self-Worth by Appearance

Supplementary File S5

Self-Worth by Others

Total Difficulties

Total Problem Behavior

Total Quality of Life
