# Supplementary material for: Social inequalities in the effects of school-based well-being interventions: a systematic review
Source: Eur J Public Health. 2025 Feb 20;35(2):302–11. doi: 10.1093/eurpub/ckaf005 (PMC11967906; doi:10.1093/eurpub/ckaf005)
Supplement: ckaf005_Supplementary_Data [file ckaf005_supplementary_data.zip › ckaf005_Supplementary_Data/ejph-2024-05-om-0341-File009.pdf]

Supplementary File S6

Quality assessment of included studies by using Effective Public Health Practice Project tool (EPHPP) Quality Assessment Tool for Quantitative Studies.

Author, year	Selection bias	Study design	Confounders	Blinding	Data collection method	Withdrawals and dropouts	Global rating
Atkins and Hayes 2019	Weak	Strong	Strong	Weak	Strong	Weak	Weak
Bell et al. 2022	Weak	Strong	Strong	Weak	Strong	Moderate	Weak
Bolling et al. 2019	Weak	Strong	Strong	Weak	Strong	Weak	Weak
Borman et al. 2019	Moderate	Strong	Strong	Weak	Strong	Weak	Weak
Brennan et al. 2021	Weak	Strong	Weak	Weak	Strong	Weak	Weak
BunketorpKäll et al. 2015	Weak	Strong	Strong	Weak	Strong	Weak	Weak
Carroll et al. 2020	Weak	Moderate	Strong	Weak	Strong	Weak	Weak
Christiansen et al. 2018	Moderate	Strong	Strong	Weak	Strong	Strong	Moderate
Costigan et al. 2016	Weak	Strong	Strong	Moderate	Strong	Strong	Moderate
Diao et al. 2020	Strong	Strong	Strong	Weak	Strong	Strong	Moderate
Ford et al. 2019	Strong	Strong	Strong	Moderate	Strong	Strong	Strong
Garbett et al. 2021	Weak	Strong	Strong	Weak	Strong	Moderate	Weak
Golan et al. 2014	Weak	Moderate	Strong	Weak	Strong	Weak	Weak
Gordon et al. 2021	Weak	Strong	Strong	Weak	Strong	Weak	Weak
Harris et al. 2022	Weak	Strong	Strong	Moderate	Strong	Strong	Moderate

Supplementary File S6

Harvey et al. 2023	Weak	Strong	Strong	Weak	Strong	Strong	Weak
Ialongo et al. 2019	Weak	Strong	Strong	Weak	Strong	Strong	Weak
Iwahori et al. 2022	Moderate	Strong	Strong	Weak	Strong	Moderate	Moderate
Johnson et al. 2016	Weak	Strong	Strong	Weak	Strong	Moderate	Weak
Johnson et al. 2017	Moderate	Strong	Strong	Weak	Strong	Weak	Weak
Kiviruusu et al. 2016	Weak	Strong	Strong	Weak	Strong	Weak	Weak
Laakso et al. 2023	Weak	Strong	Strong	Weak	Strong	Weak	Weak
Lassander et al. 2021	Weak	Strong	Strong	Moderate	Strong	Weak	Weak
Lee et al. 2018	Weak	Moderate	Weak	Moderate	Strong	Strong	Weak
Li et al. 2019	Strong	Strong	Strong	Moderate	Strong	Strong	Strong
Lubans et al. 2022	Weak	Strong	Strong	Weak	Strong	Weak	Weak
Madsen et al. 2020	Moderate	Strong	Strong	Weak	Strong	Weak	Weak
Magalhães et al. 2022	Weak	Strong	Strong	Weak	Strong	Strong	Weak
Montero-Marin et al. 2022	Moderate	Strong	Strong	Weak	Strong	Strong	Strong
Olive et al. 2019	Moderate	Strong	Strong	Moderate	Strong	Moderate	Strong
Peltonen et al. 2022	Moderate	Strong	Weak	Weak	Strong	Weak	Weak
Pollak et al. 2023	Weak	Strong	Weak	Weak	Strong	Weak	Weak
Shinde et al. 2018	Strong	Strong	Strong	Moderate	Strong	Moderate	Strong

Supplementary File S6

Shoshani and Steinmetz 2014	Weak	Strong	Weak	Weak	Strong	Strong	Weak
Shoshani et al. 2016	Strong	Strong	Strong	Moderate	Strong	Strong	Strong
Skoradal et al. 2023	Weak	Strong	Strong	Weak	Strong	Weak	Weak
Smith et al. 2018	Moderate	Strong	Strong	Weak	Strong	Strong	Moderate
Spence et al. 2014	Weak	Strong	Strong	Weak	Strong	Weak	Weak
Stjernqvist et al. 2018	Weak	Strong	Strong	Weak	Strong	Strong	Weak
Streimann et al. 2020	Weak	Strong	Strong	Weak	Strong	Strong	Weak
Torok et al. 2019	Moderate	Moderate	Weak	Weak	Strong	Weak	Weak
Troncoso and Humphrey 2021	Strong	Strong	Strong	Weak	Strong	Weak	Weak
Volkaert et al. 2022	Weak	Strong	Strong	Weak	Strong	Strong	Weak
Åvitsland et al. 2020	Weak	Strong	Strong	Weak	Strong	Weak	Weak
