# Supplementary material for: Social inequalities in the effects of school-based well-being interventions: a systematic review
Source: Eur J Public Health. 2025 Feb 20;35(2):302–11. doi: 10.1093/eurpub/ckaf005 (PMC11967906; doi:10.1093/eurpub/ckaf005)
Supplement: ckaf005_Supplementary_Data [file ckaf005_supplementary_data.zip › ckaf005_Supplementary_Data/ejph-2024-05-om-0341-File010.pdf]

41. Diao H, Wang H, Yang L, Li T. The impacts of multiple obesity-related interventions on quality of life in children and adolescents: a randomized controlled trial. Health & Quality of Life Outcomes. 2020;18(1):1–9.
42. Ford T, Hayes R, Byford S, Edwards V, Fletcher M, Logan S, et al. The effectiveness and cost-effectiveness of the Incredible Years ® Teacher Classroom Management programme in primary school children: Results of the STARS cluster randomised controlled trial. Psychological Medicine. 2019;49(5):828–42.
43. Garbett KM, Lewis-Smith H, Chaudhry A, Shroff H, Dhillon M, White P, et al. Acceptability and preliminary efficacy of a school-based body image intervention in urban India: A pilot randomised controlled trial. Body Image. 2021 Jun;37:282–90.
44. Golan M, Hagay N, Tamir S. Gender Related Differences in Response to “In Favor of Myself” Wellness Program to Enhance Positive Self & Body Image among Adolescents. Manco M, editor. PLoS ONE. 2014 Mar 11;9(3):e91778.
45. Gordon CS, Jarman HK, Rodgers RF, McLean SA, Slater A, Fuller-Tyszkiewicz M, et al. Outcomes of a Cluster Randomized Controlled Trial of the SoMe Social Media Literacy Program for Improving Body Image-Related Outcomes in Adolescent Boys and Girls. Nutrients. 2021;13(11).
46. Harris N, Warbrick I, Fleming T, Borotkanics R, Atkins D, Lubans D. Impact of high‐intensity interval training including Indigenous narratives on adolescents’ mental health: a cluster‐randomised controlled trial. Australian and New Zealand Journal of Public Health. 2022 Dec;46(6):794–9.
47. Harvey LJ, White FA, Hunt C, Abbott M. Investigating the efficacy of a Dialectical behaviour therapy-based universal intervention on adolescent social and emotional well-being outcomes. Behaviour Research and Therapy. 2023 Oct;169:104408.
48. Ialongo NS, Domitrovich C, Embry D, Greenberg M, Lawson A, Becker KD, et al. A randomized controlled trial of the combination of two school-based universal preventive interventions. Developmental Psychology. 2019 Jun;55(6):1313–25.
49. Iwahori M, Oshiyama C, Matsuzaki H. A quasi-experimental controlled study of a school-based mental health programme to improve the self-esteem of primary school children. Humanit Soc Sci Commun. 2022 Apr 27;9(1):148.
50. Johnson C, Burke C, Brinkman S, Wade T. Effectiveness of a school-based mindfulness program for transdiagnostic prevention in young adolescents. Behav Res Ther. 2016;81:1–11.
51. Johnson C, Burke C, Brinkman S, Wade T. A randomized controlled evaluation of a secondary school mindfulness program for early adolescents: Do we have the recipe right yet? Behav Res Ther. 2017;99:37–46.
52. Kiviruusu O, Björklund K, Koskinen HL, Liski A, Lindblom J, Kuoppamäki H, et al. Short-term effects of the “Together at School” intervention program on children’s socio-emotional skills: a cluster randomized controlled trial. BMC Psychology. 2016 May 26;4(1):27.
53. Laakso M, Fagerlund Å, Pesonen AK, Figueiredo RAO, Eriksson JG. The Impact of the Positive Education Program Flourishing Students on Early Adolescents’ Daily Positive and Negative Emotions Using the Experience Sampling Method. The Journal of Early Adolescence. 2023 Apr;43(4):385–417.
54. Lassander M, Hintsanen M, Suominen S, Mullola S, Vahlberg T, Volanen SM. Effects of school-based mindfulness intervention on health-related quality of life: moderating effect of gender, grade, and independent practice in cluster randomized controlled trial. Qual Life Res. 2021 Dec;30(12):3407–19.
55. Lee GY, Park EJ, Kim YR, Kwag KH, Park JH, An SH, et al. Feasibility and acceptability of a prevention program for eating disorders (Me, You and Us) adapted for young adolescents in Korea. Eat Weight Disord. 2018 Oct;23(5):673–83.
56. Li B, Pallan M, Liu WJ, Hemming K, Frew E, Lin R, et al. The CHIRPY DRAGON

intervention in preventing obesity in Chinese primary-school--aged children: A cluster-randomised controlled trial. PLoS Medicine. 2019;16(11):1–20.
57. Lubans DR, Sanders T, Noetel M, Parker P, McKay H, Morgan P, et al. Scale-up of the Internet-based Professional Learning to help teachers promote Activity in Youth (iPLAY) intervention: a hybrid type 3 implementation-effectiveness trial. Int J Behav Nutr Phys Act. 2022 Dec 1;19(1):141.
58. Madsen M, Elbe A, Madsen EE, Ermidis G, Ryom K, Wikman JM, et al. The ‘11 for Health in Denmark’ intervention in 10- to 12-year-old Danish girls and boys and its effects on well-being—A large-scale cluster RCT. Scandinavian Journal of Medicine & Science in Sports. 2020;30(9):1787–95.
59. Magalhães S, Nunes T, Soeiro I, Rodrigues R, Coelho A, Pinheiro M, et al. A Pilot Study Testing the Effectiveness of a Mindfulness-Based Program for Portuguese School Children. Mindfulness. 2022 Nov;13(11):2751–64.
60. Montero-Marin J, Allwood M, Ball S, Crane C, De Wilde K, Hinze V, et al. School-based mindfulness training in early adolescence: what works, for whom and how in the MYRIAD trial? Evid Based Mental Health. 2022 Aug;25(3):117–24.
61. Olive LS, Byrne D, Cunningham RB, Telford RM, Telford RD. Can physical education improve the mental health of children? The LOOK study cluster-randomized controlled trial. Journal of Educational Psychology. 2019 Oct;111(7):1331–40.
62. Peltonen K, Aalto S, Vänskä M, Lepistö R, Punamäki RL, Soye E, et al. Effectiveness of Promotive and Preventive Psychosocial Interventions on Improving the Mental Health of Finnish-Born and Immigrant Adolescents. International Journal of Environmental Research and Public Health. 2022 Jan;19(6):3686.
63. Pollak I, Stiehl KAM, Birchwood J, Schrank B, Zechner KA, Wiesner C, et al. Promoting Peer Connectedness Through Social-Emotional Learning: Evaluating the Intervention Effect Mechanisms and Implementation Factors of a Social-Emotional Learning Programme for 9 to 12-Year-Olds. J Youth Adolescence [Internet]. 2023 Oct 5 [cited 2023 Nov 23]; Available from: https://link.springer.com/10.1007/s10964-023-01871-x
64. Shinde S, Weiss HA, Varghese B, Khandeparkar P, Pereira B, Sharma A, et al. Promoting school climate and health outcomes with the SEHER multi-component secondary school intervention in Bihar, India: a cluster-randomised controlled trial. The Lancet. 2018 Dec;392(10163):2465–77.
65. Shoshani A, Steinmetz S. Positive Psychology at School: A School-Based Intervention to Promote Adolescents’ Mental Health and Well-Being. J Happiness Stud. 2014 Dec 1;15(6):1289–311.
66. Shoshani A, Steinmetz S, Kanat-Maymon Y. Effects of the Maytiv positive psychology school program on early adolescents’ well-being, engagement, and achievement. J Sch Psychol. 2016;57:73–92.
67. Skoradal M, Olsen MH, Madsen M, Larsen MN, Mohr M, Krustrup P. “11 for Health” in the Faroe Islands: Popularity in schoolchildren aged 10–12 and the effect on well-being and health knowledge. Scandinavian Med Sci Sports. 2023 Jun;33(6):1010–20.
68. Smith JJ, Beauchamp MR, Faulkner G, Morgan PJ, Kennedy SG, Lubans DR. Intervention effects and mediators of well-being in a school-based physical activity program for adolescents: The ‘Resistance Training for Teens’ cluster RCT. Mental Health and Physical Activity. 2018 Oct;15:88–94.
69. Spence S, Sawyer M, Sheffield J, Patton G, Bond L, Graetz B, et al. Does the Absence of a Supportive Family Environment Influence the Outcome of a Universal Intervention for the Prevention of Depression? IJERPH. 2014 May 13;11(5):5113–32.
70. Stjernqvist NW, Sabinsky M, Morgan A, Trolle E, Thyregod C, Maindal HT, et al. Building school-based social capital through ‘We Act - Together for Health’ – a quasi-experimental study. BMC Public Health. 2018 Dec;18(1):1141.

71. Streimann K, Selart A, Trummal A. Effectiveness of a Universal, Classroom-Based Preventive Intervention (PAX GBG) in Estonia: a Cluster-Randomized Controlled Trial. Prev Sci. 2020 Feb;21(2):234–44.
72. Torok M, Rasmussen V, Wong Q, Werner-Seidler A, O'Dea B, Toumbourou J, et al. Examining the impact of the Good Behaviour Game on emotional and behavioural problems in primary school children: A case for integrating well-being strategies into education. Australian Journal of Education. 2019 Nov;63(3):292–306.
73. Troncoso P, Humphrey N. Playing the long game: A multivariate multilevel non-linear growth curve model of long-term effects in a randomized trial of the Good Behavior Game. Journal of School Psychology. 2021 Oct;88:68–84.
74. Volkaert B, Wante L, Loeys T, Boelens E, Braet C. The Evaluation of Boost Camp: A Universal School-Based Prevention Program Targeting Adolescent Emotion Regulation Skills. School Mental Health. 2022 Jun;14(2):440–53.
75. Åvitsland A, Leibinger E, Resaland GK, Solberg RB, Kolle E, Dyrstad SM. Effects of school-based physical activity interventions on mental health in adolescents: The School in Motion cluster randomized controlled trial. Mental Health and Physical Activity. 2020 Oct 1;19:100348.
76. Love R, Adams J, van Sluijs EMF. Are school-based physical activity interventions effective and equitable? A meta-analysis of cluster randomized controlled trials with accelerometer-assessed activity. Obesity Reviews. 2019;20(6):859–70.
77. Cole ER, Duncan LE. Better policy interventions through intersectionality. Social Issues and Policy Review. 2023;17(1):62–78.
78. Sun X, Ioannidis JPA, Agoritsas T, Alba AC, Guyatt G. How to Use a Subgroup Analysis: Users' Guide to the Medical Literature. JAMA. 2014 Jan 22;311(4):405–11.
79. Berkman LF, Avendano M, Courtin E. Producing Change to Understand the Social Determinants of Health: The Promise of Experiments for Social Epidemiology. American Journal of Epidemiology. 2022 Aug 9;kwac142.
80. Fischer C, Fishman B, Levy AJ, Eisenkraft A, Dede C, Lawrenz F, et al. When Do Students in Low-SES Schools Perform Better-Than-Expected on a High-Stakes Test? Analyzing School, Teacher, Teaching, and Professional Development Characteristics. Urban Education. 2020 Oct;55(8–9):1280–314.
81. Mansfield KL, Ukoumunne OC, Blakemore SJ, Montero-Marin J, Byford S, Ford T, et al. Missing the context: The challenge of social inequalities to school-based mental health interventions. JCPP Advances. 2023;3(2):e12165.
